# Supplementary material for: Doping-Dependent Optical Response of a Hybrid Transparent Conductive Oxide/Plasmonic Medium
Source: J Phys Chem C Nanomater Interfaces. 2022 Jan 25;126(4):1881–9. doi: 10.1021/acs.jpcc.1c07567 (PMC8819857; doi:10.1021/acs.jpcc.1c07567)
Supplement: Supplementary file 1 — jp1c07567_si_001.pdf [file jp1c07567_si_001.pdf]

Doping-Dependent Optical Response of a Hybrid Transparent Conductive Oxide/Plasmonic Medium

Maria Sygletou,*,† Stefania Benedetti,‡ Alessandro di Bona,‡ Maurizio Canepa,† and Francesco Bisio¶

†*OptMatLab, Dipartimento di Fisica, Università di Genova, via Dodecaneso 33, I-16146 Genova, Italy*

‡*CNR-Istituto Nanoscienze, via Campi 213/a, 41125 Modena, Italy*

¶*CNR-SPIN, C.so Perrone 24, I-16152 Genova, Italy*

E-mail: sygletou@fisica.unige.it

AFM images of bare ZnO and AZO films of different doping levels (2% and 4%), obtained in the same deposition conditions, are shown in Figure S1 (a-c). AFM images of Au NPs deposited on top of ZnO and AZO films, are also shown in Figure S1 (d-f). Representative SEM and AFM images of the parametric study for the optimization of the surface morphology and roughness of bare ZnO films on MgO substrates, by modifying the deposition parameters, are presented in Figure S2.

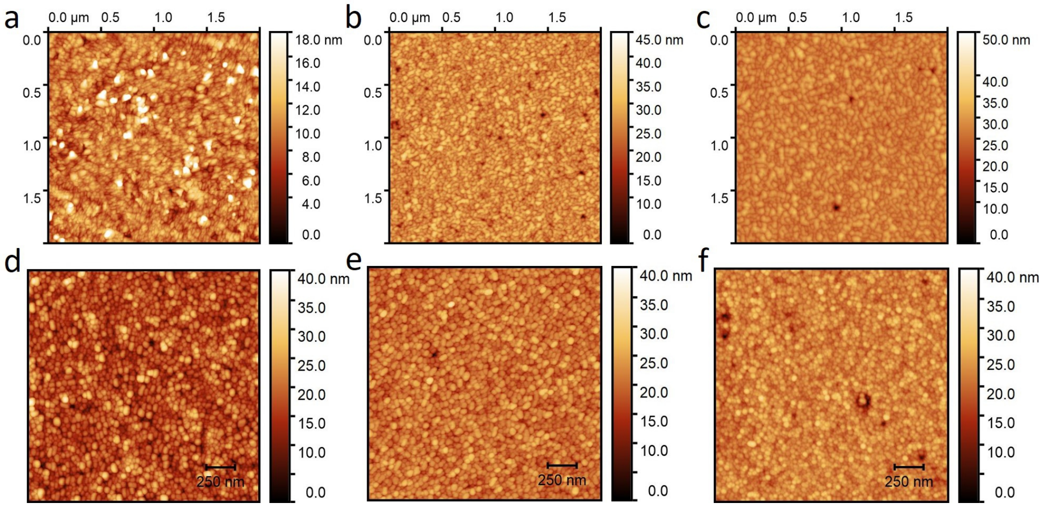

Figure S1: Top: AFM images of ZnO (a), 2 at.% AZO (b) and 4 at.% AZO (c) films of 150 nm thickness, grown on MgO substrates. Bottom: AFM images of Au NPs deposited on top of ZnO (d), 2 at.% AZO (e) and 4 at.% AZO (f) films, grown on MgO substrates.

In Figure S3 the ellipsometric spectra Ψ and Δ of bare and Al-doped (2 at.% and 4 at.%) ZnO films, acquired with incident angles of 60° and 65°, are shown, top to bottom. Green symbols correspond to experimental points, while red lines represent the best fit obtained in correspondence of the optical properties reported in Figure S4 and of the morphological parameters (film thickness, roughness) reported in the manuscript.

In Figure S5 the carriers density (N_e) and resistivity (ρ) of bare and Al-doped (2 at.% and 4 at.%) ZnO films, as extracted from SE, are reported.

The SE spectra Ψ and Δ of the Au-NP layer deposited on top of bare and Al-doped (2 at.% and 4 at.%) ZnO films, acquired with incident angles of 60° and 65°, are shown, top to bottom, in Figure S6. Green symbols correspond to experimental points, while red lines represent the best fit

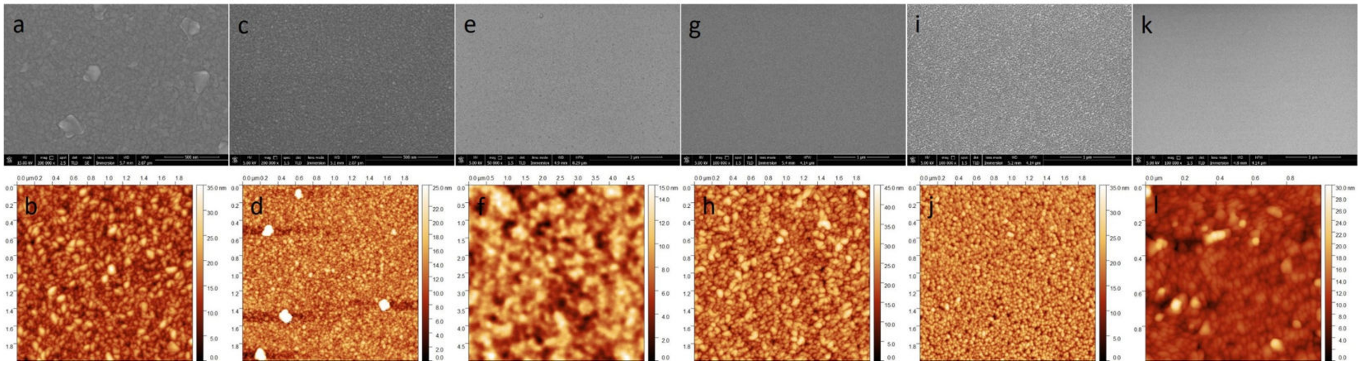

Figure S2: Typical SEM (top) and AFM (bottom) images of ZnO films on Si/SiO_2 substrate, fabricated with different deposition parameters. (a,b) ZnO film, fabricated with the deposition parameters, used as reference (Deposition of 300 nm ZnO at room temperature, in the presence of Argon, deposition rate of 1.1Å/s, pressure of 5 mTorr). In the other depositions only one parameter was changed, leaving the remaining parameters unaltered. (c,d) Deposition of ZnO film of thickness 100 nm, (e,f) deposition of ZnO film at 300°C, (g,h) deposition of ZnO film with rate of 0.5A/s and (i,j) deposition of ZnO film under the prescence of O_2. (k,l) Depostion of 100 nm ZnO film at 300°C.

obtained in correspondence of the dielectric function reported in Figure S8.

The intermix layer between ZnO (bare and Al-doped) and Au NPs is an Effective Medium Approximation (EMA) layer that models interfacial mixing or "interface" roughness by mixing the layers above (Au NPs) and below (AZO) the interface in 50:50 Bruggeman EMA. The thickness of the intermix layers between the bare and Al-doped (2 at. and 4 at. %) ZnO films and Au NPs was and 7.0 ± 0.3 nm,7.1 ± 0.1 nm and 7.6 ± 0.6 nm, respectively.

In Figure S7 we report the transmission spectra of AZO/MgO films, bare (black markers) and following the deposition of Au NPs (red markers). There we observe clearly the effect of the LSPR, which is less obvious in SE. The transparent nature in the visible region of 2 at.% AZO films fabricated in this work, is shown while the transmission dip around 600 nm in the red spectrum is the fingerprint of the LSPR.

The optical constants of Au NPs/AZO films extracted from ellipsometry were applied for the modelling of transmission measurements. The system was modelled replacing the MgO 1-side polished with a 2-side polished MgO substrate. In Figure S9(a) the experimental data from the transmission measurements are shown (green markers) along with the fit data (red lines) which are

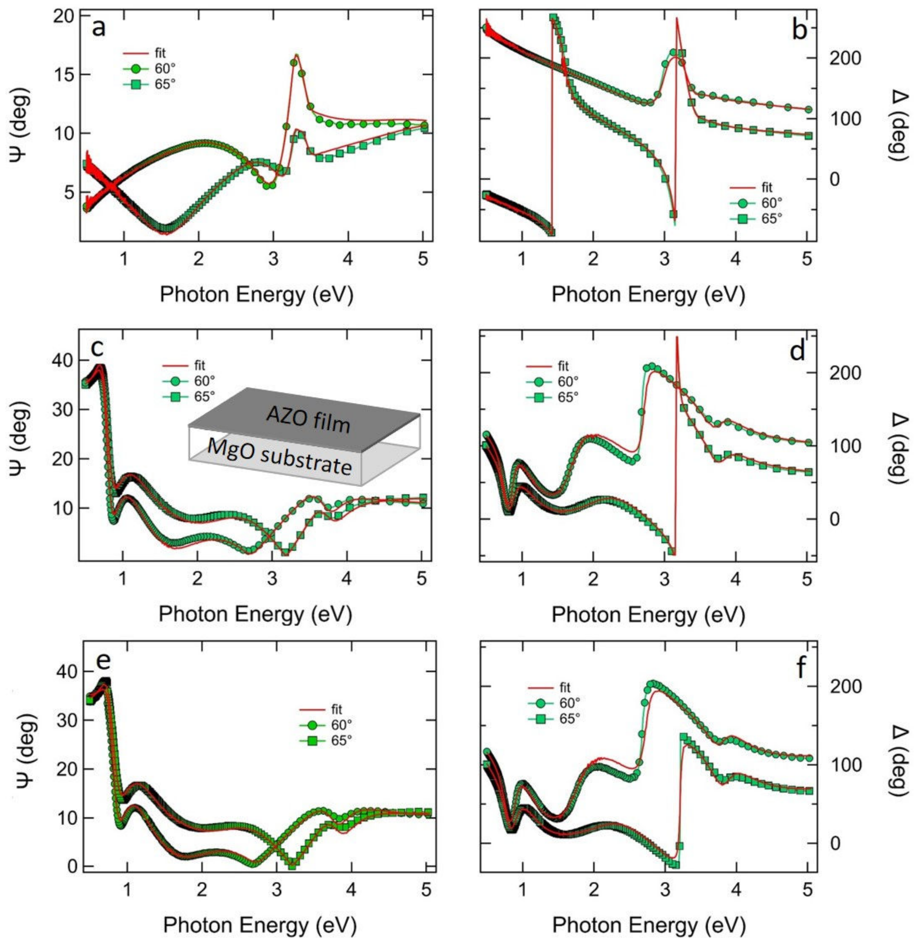

Figure S3: Ψ (left) and Δ (right) spectra of ZnO (a, b), 2 at.% AZO (c, d) and 4 at.% AZO (e, f) films, grown on MgO substrates, acquired with incident angle of 60°(circles) and 65°(squares). Lines represent the best fit to the experimental data. The inset of Figure S3(c) is a representative scheme of the samples under study (AZO film/MgO substrate, 1-side polished).

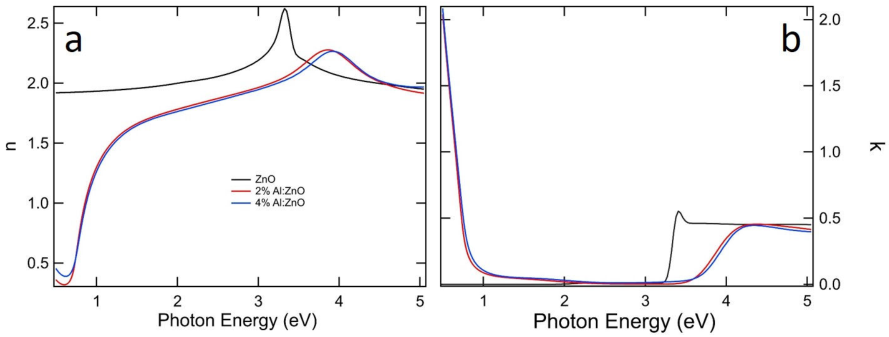

Figure S4: Refractive index, n, (a) and extinction coefficient, k, (b) of AZO films of different doping levels (2, 4% at.). The optical properties of a ZnO film (black lines) are reported for reference.

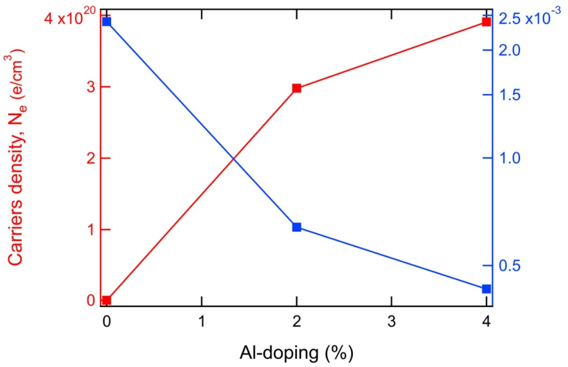

Figure S5: Carriers density (N_e) and resistivity (ρ) of bare and Al-doped ZnO films.

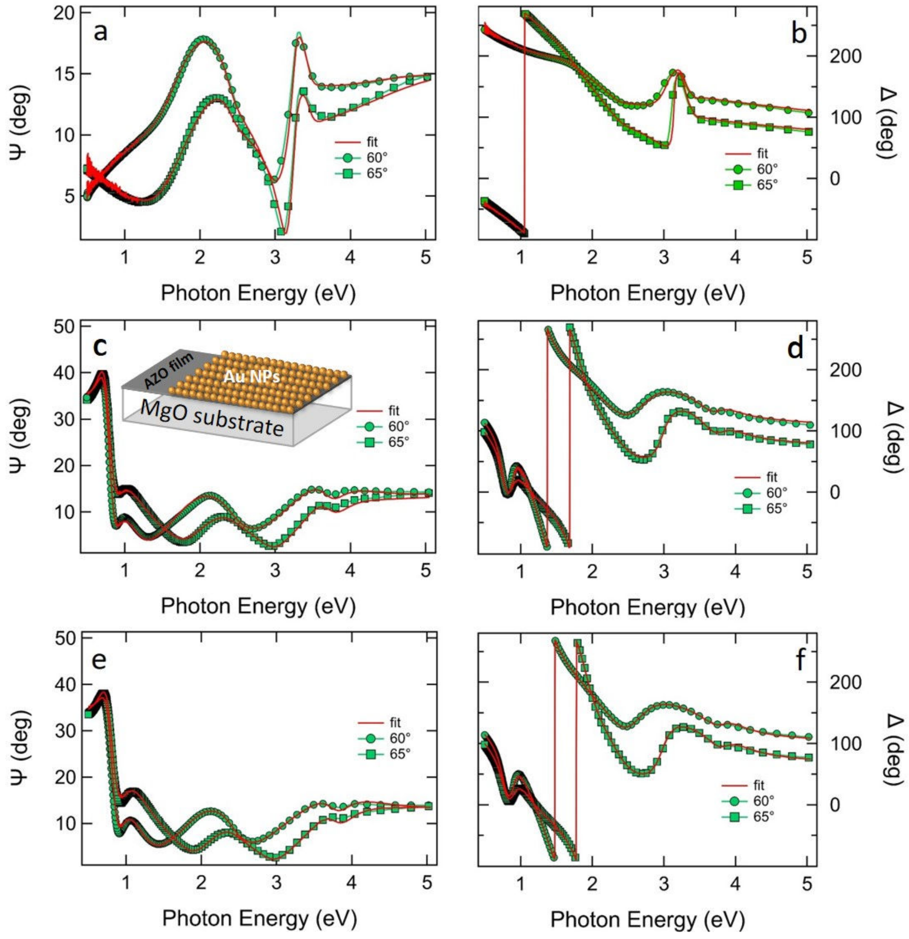

Figure S6: Ψ (left) and Δ (right) spectra of Au NPs/ZnO (a, b), Au NPs/2 at.% AZO (c, d) and Au NPs/4 at.% AZO (e, f) films, acquired with incident angle of 60°(circles) and 65°(squares). Lines represent the best fit to the experimental data. In Figures S6(c) and (d) the ellipsometric parameters Ψ and Δ of 2 at.% AZO film, acquired with incident angle of 60 ° (blue circles) are shown for comparison. The inset of Figure S6(c) is a representative scheme of the samples under study (Au NPs/AZO film/MgO, substrate 1-side polished).

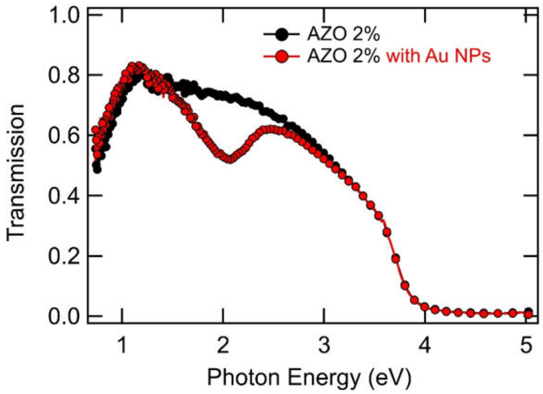

Figure S7: Transmission spectra of 2 at.% AZO films with (red markers) and without (black markers) gold NPs on top.

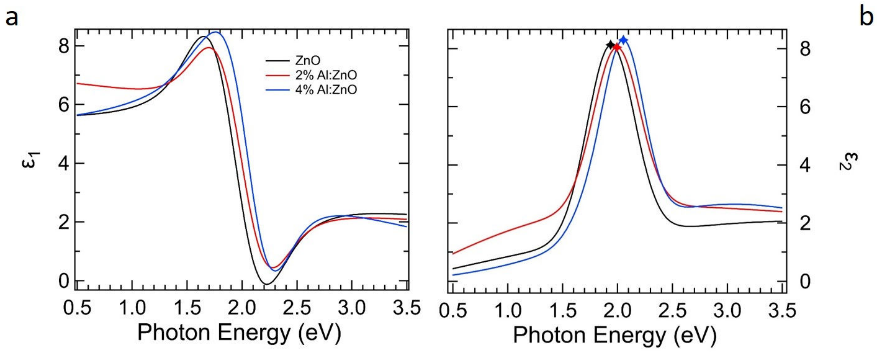

Figure S8: Real (a) and imaginary (b) part of the dielectric function of Au NPs on bare ZnO and AZO films (2 and 4 at.%), as extracted by spectroscopic ellipsometry. Markers on the ε_2 peak were placed for the sake of clarity of the LSPR blueshift.

the outcome of the modelling. The respective refractive index and extinction coefficient that came out of the modelling of this system are also shown in Figure S9.

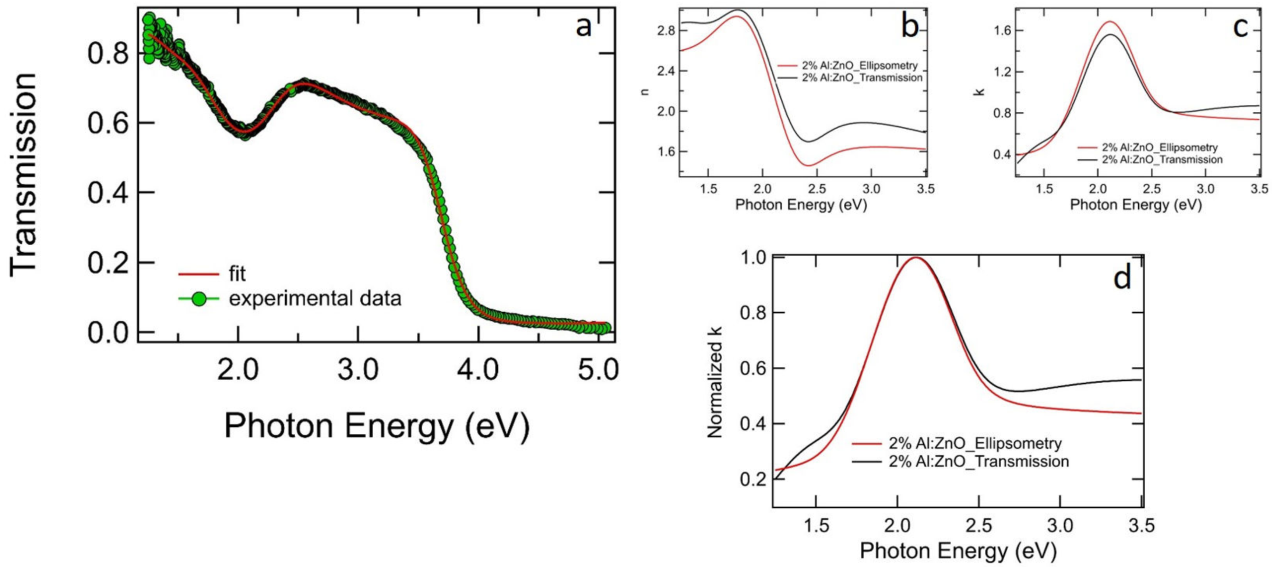

Figure S9: (a)Transmission spectrum of Au NPs on 2 at.% AZO film of 100 nm thickness, grown on 2-side polished MgO substrate. Circles represent the experimental data while lines the theoretical curve, calculated from the dielectric parameters obtained from ellipsometry measurements on 1-side polished MgO substrate as in Figure 5 of the manuscript. Effective refractive index, n, (b) and extinction coefficient, k, (c) of the Au-NP layer deposited on the 2 at.% AZO film on 2-side polished MgO substrate (black lines). The optical properties of Au NPs/2 at.% AZO on 1-side polished MgO (red lines) are shown for comparison. (d) Normalized extinction coefficient of the two hybrid systems.
